# Analysis of Factors of Variation in Characteristics of Boar Ejaculates

**DOI:** 10.3390/ani15142043

**Published:** 2025-07-11

**Authors:** Stanisław Kondracki, Krzysztof Górski

**Affiliations:** Institute of Animal Science and Fisheries, University of Siedlce, Prusa 14, 08-110 Siedlce, Poland; stanislaw.kondracki@uws.edu.pl

**Keywords:** boar, traits of ejaculate, variation, breed, age, season

## Abstract

This study aims to analyse the effect of selected variation factors on the ejaculate characteristics of boars and to characterise changes in ejaculate characteristics in Landrace, Large White, Duroc, and Pietrain boars during their use for artificial insemination. The original value of this work lies in the estimation of the percentage share of individual components of variability in shaping the traits of boar ejaculate. A total of 943 ejaculates collected from 77 boars used for artificial insemination were analysed. This study began when the boars were at 8–9 months old. Ejaculates were collected in nine consecutive months from the start of the boars’ use. Immediately after collection, they were analysed for ejaculate volume, sperm concentration, percentage of sperm with progressive motility, total number of spermatozoa, and number of insemination doses per ejaculate. The results were analysed according to three criteria: breed of boar (Landrace, Large White, Duroc, and Pietrain), age of boar (up to 10 months, 11–13 months, 14–17 months, and more than 17 months), and season (spring, summer, autumn, and winter). The analysis of the variation in ejaculate characteristics took into account the share of each factor (boar breed, boar age, and season) in the variation, as well as the interactions between factors. The effects of the three factors and interactions between them were calculated using an ANOVA (analysis of variance). The variation was shown to depend mainly on the breed and age. These two factors and the interaction between them determine about 80% of the variation in ejaculate characteristics. The season also has an effect, but its share in the influence of variation on ejaculate characteristics is relatively small. Ejaculates from Landrace boars are the most favourable for insemination, with a large volume, a relatively high sperm concentration, and the highest number of sperm. The highest number of insemination doses can be prepared from Landrace ejaculates—on average, 2.7–6.7 more doses than from the other breeds. Duroc boar ejaculates are most distinctive, with a very low volume but a very high sperm concentration and the highest sperm motility. The ejaculates of Pietrain boars showed the opposite pattern, with the largest volume but the lowest sperm concentration. The sexual development of young boars, expressed as an increase in ejaculation performance, progresses during their first year of insemination use.

## Simple Summary

The results of this study clearly show that variations in boar ejaculate characteristics mainly depend on breed and age. The season of the year also has an effect, but its share in the influence of variation on ejaculate characteristics is relatively small. Ejaculates produced by Landrace boars are the most favourable for insemination. The ejaculates of Duroc boars are the most distinctive, with a very low volume in addition to a very high sperm concentration and the highest sperm motility, in contrast to Pietrain boars, whose ejaculates have a larger volume but a low sperm concentration. The rate of the sexual development of young boars depends on their breed. It is highest in Pietrain and Landrace boars and much lower in Large White boars, while changes in ejaculate characteristics in Duroc boars in the first year of use are very minor.

## 1. Introduction

The ejaculates of boars used for artificial insemination are highly variable. Sources of variation in ejaculates include genotype [1,2], season [3,4], and organisational factors [5,6]. Individual variation plays an important role as well. It has been demonstrated that some individuals are more predisposed than others for use for artificial insemination, and that there is high individual variation within a given breed [7,8]. Boars selected for commercial use are evaluated for fattening and carcass traits [9,10]. In addition to these traits, boars used for artificial insemination should also have traits predisposing them to breeding, and thus a high libido and the ability to produce a large amount of high-quality semen [6,11,12].

Studies of the ejaculates of males used for artificial insemination often take into account the effect of the boar’s age and breed and the season of the year on ejaculate characteristics [13,14]. However, there have been few studies investigating the shares and proportions of these three factors and the interactions between them in determining the variation in ejaculate characteristics. In practice, it is difficult to determine which of the many sources of variation are most important for the male’s suitability for artificial insemination—whether it is variation resulting from genetic effects, such as the breed, crossbreeding variant, or level of heterozygosity, or variation resulting from environmental factors, such as the type and level of feeding, housing conditions, and season. Also unknown is the share of the total variation determined by the effects of factors associated with the animal itself, such as age and specific predispositions for artificial insemination, and the share of the variation determined by organisational effects, resulting from how the breeder is used. However, no studies have been conducted that would determine the impact of the boar’s breed and age as well as the season on ejaculate characteristics, within a single experiment.

The aim of this study is to analyse the effect of selected factors of variation on the ejaculate characteristics of boars and to characterise the changes in ejaculate characteristics in Landrace, Large White, Duroc and Pietrain boars during their use for artificial insemination.

## 2. Materials and Methods

### 2.1. Animals and Semen Collection

This study was carried out in strict compliance with the recommendations in Directive 63/2010/EU and the Journal of Laws of the Republic of Poland of 2015 on the protection of animals used for scientific or educational purposes. This study was approved by the Polish Laboratory Animal Science Association (Number 3401/2015).

The physical characteristics of 943 ejaculates collected from 77 boars used for artificial insemination were evaluated. Young insemination boars at the age of 8–9 months were selected for the analysis. This study was conducted on boars used at a commercial insemination station located in central Poland. Boars were kept individually in pens with an area of 8 m^2^, with straw bedding and nipple drinkers. The average air temperature in the buildings for boars was 15 °C (min. 12 °C, max. 20 °C), and the average relative humidity was 75% (min. 65%, max. 85%). Air movement within the buildings was 0.15 m/s in winter and 0.2 m/s in summer. Temperature and humidity were recorded using a thermo-hygrometer TERMIK PLUS (100029, Termoprodukt, Bielawa, Poland, PL). Temperature was measured with a precision of one degree Celsius. Humidity, expressed as a percentage, was measured with a precision of one percent point. The temperature and humidity sensors were placed in the central part of the piggery, halfway in the aisle dividing the facility into two equal parts. The air velocity was measured with a thermal anemometer TA35 (Airflow^TM^, High Wycombe, UK). The outdoor mean ambient temperature and humidity per season were 3.1 °C and 85.6% (winter), 13.6 °C and 59.2% (spring), 24.7 °C and 58.0% (summer), and 12.8 °C and 78.2% (autumn), respectively. The boars selected for the study were in good health, with no evident developmental defects and with a normal libido. The boars were used for artificial insemination, and ejaculate was collected every four days. Ejaculates were collected manually using the gloved-hand technique. Semen was collected using a clean semen collecting flask that filters out gel, dust, and bristles [15]. Assessments were carried out on ejaculates collected in nine consecutive months from the start of the boar’s use (8–9 months of age).

### 2.2. Semen Evaluation

Immediately after collection, the ejaculates were analysed for the following: ejaculate volume, sperm concentration, percentage of sperm with progressive motility, total number of spermatozoa, and number of insemination doses per ejaculate. Ejaculate volume was determined after separating the gel fraction based on the measurement of the ejaculate weight with an electronic balance. Sperm concentration in the ejaculate was determined via photometry using a SPERMACUE electronic photometer (Minitube, GmbH, Tiefenbach, Germany). This method involves measuring the intensity of light passing through a microcuvette containing an undiluted semen sample at a path length of 0.7 µm. Sperm motility was determined through microscope examination at 200× magnification, and the percentage of sperm exhibiting progressive motility in the total number of sperm in the field of view of the microscope was calculated. The total number of spermatozoa in the ejaculate, the number of insemination doses per ejaculate, and the number of sperm per insemination dose were calculated using SYSTEM SUL computer software (v.6.3.5).

### 2.3. Experimental Design

The material was grouped according to three criteria: boar breed, boar age, and the season when the ejaculate was collected.

Effect of boar:

Landrace (31 boars)

Large White (30 boars)

Duroc (8 boars)

Pietrain (8 boars)

Effect of season:

Spring—ejaculates collected in March, April and May

Summer—ejaculates collected in June, July, and August

Autumn—ejaculates collected in September, October, and November

Winter—ejaculates collected in December, January, and February

Effect of boar age:

Group I—ejaculates collected from boars up to and including 10 months of age

Group II—ejaculates collected from boars at the age of 11 to 13 months

Group III—ejaculates collected from boars at the age of 14 to 17 months

Group IV—ejaculates collected from boars above 17 months of age

The experimental data were subjected to a statistical analysis with STATISTICA software (version 13.1 PL, StatSoft, Tulsa, OK, USA), using analysis of variance ANOVA for different N (N = number of subjects in groups). Variation in semen characteristics was analysed according to the following mathematical model:

Y*_ijkl_* = µ + a*_i_* + b*_j_* + c*_k_* + ab*_ij_* + ac*_ik_* + bc*_jk_* + abc*_ijk_* + e*_ijkl_* (1)

where

Y*_ijkl_*—value of characteristics

µ—grand mean

a*_i_*—effect of boar breed

b*_j_*—effect of boar age

c*_k_*—effect of season

ab*_ij_*, ac*_ik_*, bc*_jk_*, abc*_ijk_*—effect of interaction of factors

e*_ijkl_*—error

Significance of differences between groups was determined by Tukey’s test at *p* < 0.05.

The analysis of the variation in ejaculate characteristics was based on the share of mean square deviations for the sources of variation (breed, age, and season), for the interactions between factors, and for the error in the total variation in a given characteristic. This made it possible to estimate the proportion of the variation in ejaculate characteristics resulting from the effect of the individual factors tested in the experiment. Models of the variation in ejaculate characteristics of boars were also constructed separately for each of the four breeds in relation to the age of the boar.

## 3. Results

### 3.1. Structure of Variation Sources

Table 1 presents data enabling the determination of the percentage share of individual components of variation in the total variation in the ejaculate characteristics of boars.

**Figure 1:**
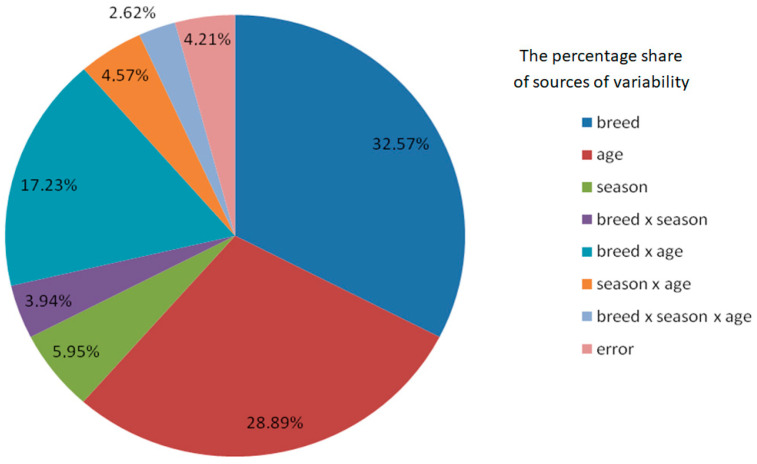
Percentage share of individual sources of variation in determining the number of sperm in the ejaculate.

The data in Table 1 can be used to determine the effect of the boar’s breed and age and the season when the ejaculates were collected on their quality. The data clearly indicate that ejaculate characteristics were most dependent on the breed of the boar. Variation in ejaculate characteristics resulting from breed constituted from 13.13% of the total variation in the case of sperm motility to 40.82% in the case of sperm concentration. Another important source of variation in ejaculate characteristics was the age of the boar, particularly in the case of ejaculate volume (35.73% of total variation) and total number of spermatozoa (28.89% of total variation). Moreover, the age of the boar had a relatively small role in determining the variation in sperm concentration (3.99% of total variation) and sperm motility (9.02% of total variation). The data in Table 1 show that the role of the season of the year in determining ejaculate characteristics was markedly smaller than the effect of the breed and age of the boar. In most cases it did not exceed 10%, ranging from 4.97% for sperm concentration to 12.16% for ejaculate volume. The suitability of boar ejaculate depends to the greatest extent on the number of sperm with progressive motility. Many insemination doses can be prepared from ejaculates with a high content of motile sperm. The structure of the factors of variation for the number of sperm with progressive motility is presented in Figure 1.

The structure of the sources of variation in the total number of spermatozoa shows that nearly 80% of the variation in this trait results from the interaction of two factors: the breed and age of the boar (effect of breed—32.57%; effect of age—28.89%; breed × age interaction—17.23% of total variation). The remaining sources of variation play a small role in determining the total number of spermatozoa (none of them accounted for more than 6% of the total variation).

### 3.2. Effect of Breed

The analysis of the effect of breed took into account four breeds of boars: Landrace, Large White, Pietrain and Duroc (Table 2).

The data in Table 2 document the occurrence of significant and in some cases large differences in the characteristics of ejaculates obtained from boars of different breeds. Duroc boar ejaculates were the most distinctive, with a very small ejaculate volume that was 58–86 mL smaller than in the other breeds (*p* < 0.05), and at the same time a very high sperm concentration (529.23 × 10^6^/mL), which was 73–118 × 10^6^/mL higher than in the remaining breeds (*p* < 0.05). Duroc boars were also distinguished by the highest sperm motility. The opposite pattern was shown in the ejaculates of Pietrain boars, which had the highest ejaculate volume and the lowest sperm concentration.

The ejaculates of Landrace boars were the most favourable for artificial insemination. They had a relatively high volume with a relatively high sperm concentration, and they contained the most sperm with progressive motility, about 5.4–13.7 × 10^6^/mL more than ejaculates from other breeds (*p* < 0.05). On average, more than 31 insemination doses could be prepared from the ejaculates of Landrace boars, which is 2.7–6.7 insemination doses more than from the ejaculates of boars of other breeds.

### 3.3. Effect of Age

Table 3 presents data enabling the analysis of changes in basic ejaculate characteristics depending on the age of the boar.

The data clearly indicate that ejaculate volume and the total number of spermatozoa increase with the age of the boar, resulting in an increase in the number of insemination doses per ejaculate. These changes are very pronounced, and their direction is very clear. At the start of the boars’ use for artificial insemination (at less than 10 months of age), the average ejaculate volume was only 213.92 mL, but it increased systematically with the age of the boars. Once the boar was over 17 months old, the average ejaculate volume amounted to 66.46 mL, an increase of more than 30% (*p* < 0.05). The total number of spermatozoa increased by 22.11 × 10^9^ during this period (from 74.04 × 10^9^ to 96.57 × 10^9^), i.e., by nearly 30% (*p* < 0.05). The average number of insemination doses per ejaculate collected from boars at the age of over 17 months was 31.84, which was 6.83 (over 27%) more than for ejaculates collected at the age of under 10 months.

The data show that the sexual development of young boars progresses during the first year of use. This development is manifested in an increase in ejaculation performance. Ejaculate volume and the number of sperm per ejaculate increase with ejaculation performance; thus, they increase rapidly. However, it is worth noting that the increase in quantitative characteristics is not accompanied by a similar increase in sperm concentration and sperm motility, as the data in Table 3 show that neither sperm concentration nor sperm motility changed significantly during the boars’ first year of use for artificial insemination.

**Figure 2:**
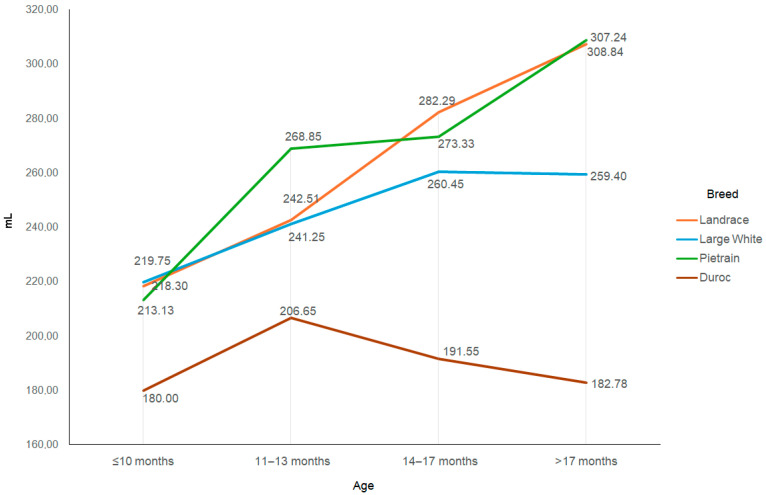
Ejaculate volume depending on the breed and age of the boar.

Changes in ejaculate characteristics in the first year of use did not proceed in the same manner in all breeds. Ejaculate volume increased very rapidly in Pietrain and Landrace boars (Figure 2), from just under 220 mL before the age of 10 months to nearly 310 mL at the age of over 17 months, i.e., by more than 40% (*p* < 0.05). The ejaculate volume of Large White boars increased at a much slower rate from about 220 mL to nearly 260 mL (*p* < 0.05), and that of Duroc boars changed very little (180–206 mL) (*p* < 0.05).

**Figure 3:**
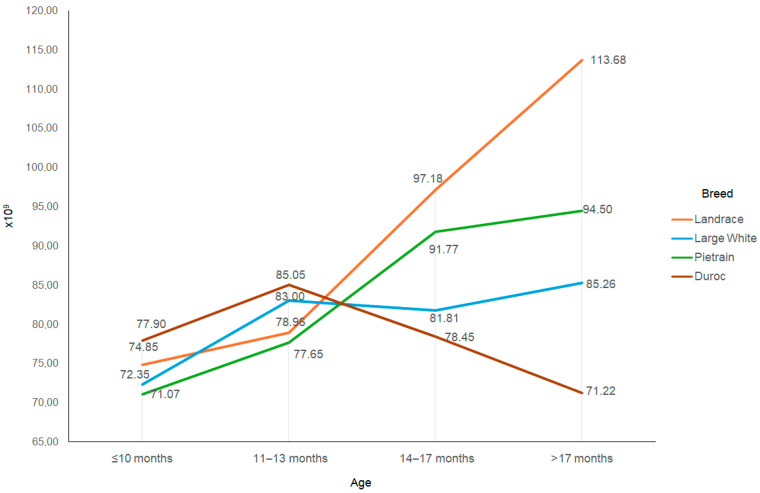
Total number of spermatozoa depending on the breed and age of the boar.

Even greater differences between breeds were observed for the number of spermatozoa in the ejaculate (Figure 3). The rate of change in the number of spermatozoa in the ejaculate was greatest in Landrace boars, in which it increased from about 75 billion at the age of under 10 months to about 114 billion in boars above the age of 17 months, i.e., by more than 50% (*p* < 0.05). The number of sperm in the ejaculates increased at a much lower rate in Pietrain boars, from about 71 billion to about 95 billion (*p* < 0.05), as well as in Large White boars, from about 72 billion to about 85 billion (*p* < 0.05). The number of spermatozoa in the ejaculates of Duroc boars showed little change and even decreased somewhat (*p* > 0.05).

**Figure 4:**
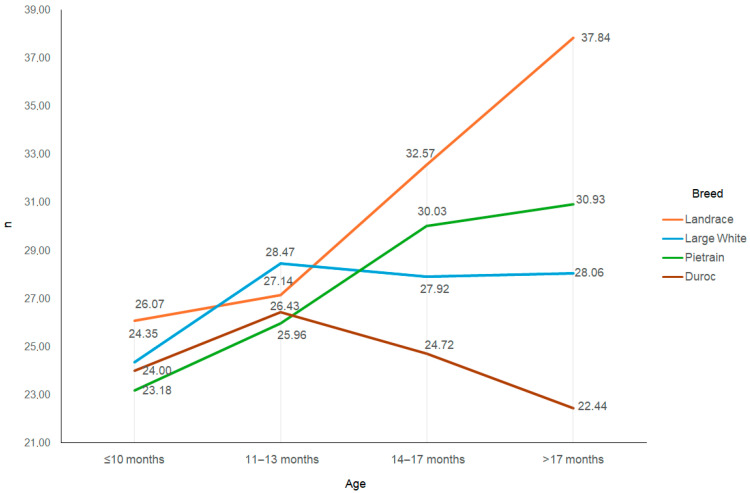
Number of insemination doses per ejaculate depending on the breed and age of the boar.

Changes in ejaculate volume and the number of spermatozoa in the ejaculates affect the number of insemination doses obtained (Figure 4). The number of insemination doses produced from the ejaculates of Landrace boars increased by more than 11 during the period analysed (*p* < 0.05), increasing by nearly 8 in Pietrain boars (*p* < 0.05) but by less than 4 in Large White boars (*p* < 0.05). The number of insemination doses per Duroc boar ejaculate did not increase during this period and even decreased somewhat.

**Figure 5:**
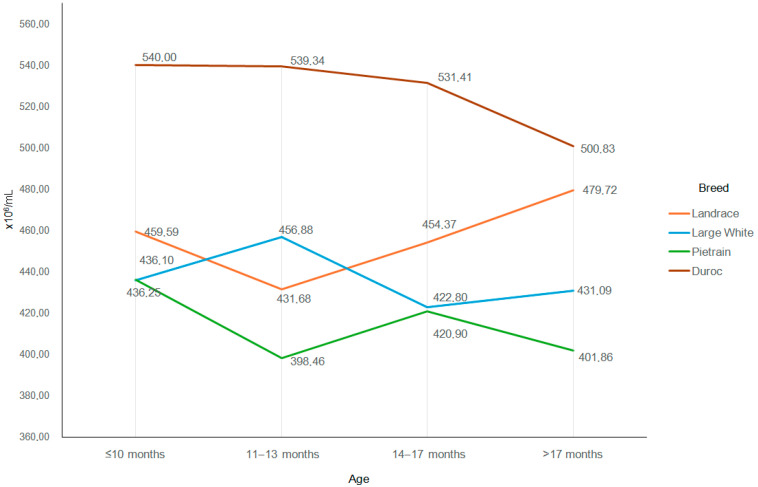
Sperm concentration depending on the breed and age of the boar.

**Figure 6:**
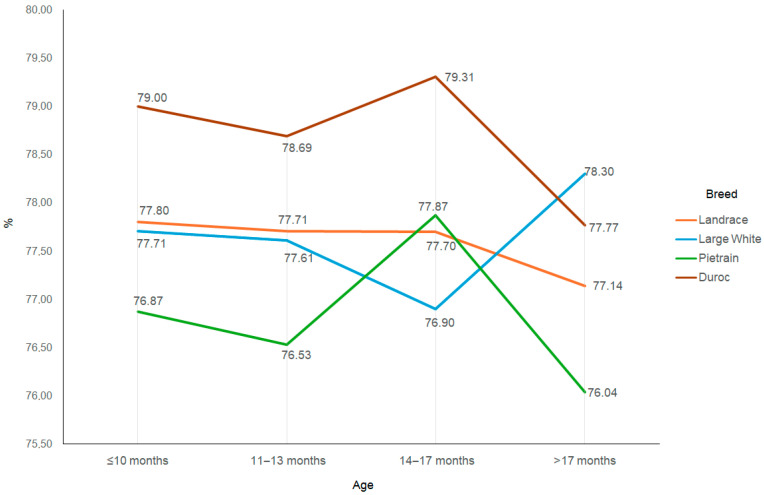
Sperm motility depending on the breed and age of the boar.

Changes in sperm concentration and sperm motility in the ejaculates collected during the study period were small (Figure 5 and Figure 6), and no differences between breeds were observed (*p* > 0.05).

### 3.4. Effect of Season

Table 4 presents data enabling the analysis of the basic characteristics of ejaculates collected in spring, summer, autumn, and winter.

These data show a minor effect of the season of the year on ejaculate characteristics. No differences are shown for sperm concentration, sperm motility, the total number of spermatozoa in the ejaculate, or the number of insemination doses per ejaculate between ejaculates collected in autumn, winter, spring, and summer. Autumn appears to be the most favourable season for the use of boars for artificial insemination. Ejaculates collected in autumn had the greatest volume, which was significantly greater than that of ejaculates collected in spring and summer (*p* < 0.05). However, the number of insemination doses per ejaculate was not affected (*p* < 0.05).

## 4. Discussion

The data presented in this study clearly demonstrate that the primary factor determining the variation in ejaculate characteristics is the breed of boar. The effect of breed accounted for the largest share of the total variation in ejaculate characteristics, as clearly documented by the data in Table 1. The present study compared the ejaculates of boars of four breeds of major importance in Europe and around the world. Pronounced and large differences were shown between breeds for basic ejaculate characteristics. The ejaculates of boars of different genotypes often differ in volume, sperm concentration, total number of spermatozoa, and number of insemination doses per ejaculate [16]. Differences have also been shown for sperm motility and fertilisation ability [17,18]. The results of the present study show that the breeds studied are distinguished by distinct breed specificity in terms of ejaculate characteristics. The data in Table 2 show that the ejaculates of Duroc boars are the most distinct from the others, as boars of this breed produced ejaculates with a very high sperm concentration but low volume. A high sperm concentration in a low ejaculate volume is a genetically fixed and characteristic trait of the Duroc breed, as confirmed by the findings of other studies on the semen of Duroc boars [16,19,20,21,22,23]. Ejaculates with a high sperm concentration usually also have a high total number of spermatozoa and good sperm motility. However, these ejaculates are often smaller in volume than those with a low sperm concentration [24]. High negative correlations (−0.55 to −0.66) between sperm concentration and ejaculate volume have also been shown in studies on boars of other breeds [25]. However, the data in Table 2 clearly demonstrate that fewer insemination doses could be obtained per Duroc boar ejaculate than from the ejaculates of other breeds. The opposite pattern was shown for the ejaculates of Pietrain boars, which had the largest volume but the lowest sperm concentration. The comparison of the ejaculates of Duroc and Pietrain boars indicates that ejaculate volume is inversely proportional to sperm concentration. This relationship is very pronounced in the ejaculates of these breeds and is most likely due to specific breed characteristics. However, this is probably a more generalised pattern, extending beyond the characteristics of the Duroc and Pietrain breeds, as similar relationships, though less pronounced, have been observed in other studies [19,26,27,28]. The existence of this pattern explains why the ejaculate volume/sperm concentration relationship in the ejaculates of Pietrain boars is the opposite of that observed in the ejaculates of Duroc boars. The data in Table 2 show that about four more insemination doses could be prepared from the ejaculates of Pietrain boars than from Duroc ejaculates. Other studies comparing the insemination efficiency of boars of these breeds have shown that about two [29] to six [21] more insemination doses are obtained from Pietrain boar ejaculates than from Duroc boar ejaculates.

The ejaculates of Landrace boars had the most favourable characteristics, as they contained the most sperm with progressive motility, and the most insemination doses could be obtained from them (Table 2). Landrace is a breed commonly used in Europe and is known around the world. In recent years, genetic lines are becoming increasingly significant in the mass production of pigs. Landrace boars generally produce ejaculates with favourable characteristics. This is not the only study confirming the high quality of ejaculates from this breed. Other studies have presented data indicating that Landrace boars produce ejaculates with greater volume and a higher sperm concentration than Large White boars [30,31,32].

The reproductive performance of boars can also be affected by their age, which is indicated by the findings of the present study. The effect of the boar’s age accounted for a large percentage of the total variation in ejaculate characteristics, which is clearly documented by the data presented in Table 1 and Figure 1. The age of the boar has a particularly strong influence on ejaculate volume. The share of the effect of age in the variation in this trait was the largest (35.73% of the total variation), more than 11% greater than the effect of breed and more than 23% greater than the effect of season (Table 1). The observations in the present study began when the boars were about 8–9 months old, the age at which boars are first used at artificial insemination stations. At this age, they are already sexually mature, but they usually reach their full reproductive capacity at a later age. This is linked to the progressive development of the internal structure of the testes, which determines the number of sperm produced, and to the development of accessory sex glands, which promotes an increase in ejaculate volume. At this time, the number of Sertoli cells in the testes increases, which is required for sperm production [33], and the secretory functions of the accessory sex glands, whose secretions influence ejaculate volume, undergo development. Boar testicles have been shown to increase in size up to the age of 20 months [30], and the greatest increase in young boars has been shown from 2 to 6 months of age—a threefold increase [34].

The ejaculate characteristics of most boars change most rapidly up to the age of 18–19 months. The present study shows that in the interval from 8 to 19 months, there is a rapid increase in ejaculate volume and the total number of spermatozoa, resulting in an increase in the number of insemination doses per ejaculate. However, no significant changes in sperm concentration or sperm motility were observed during this time. The increase in ejaculation performance was therefore primarily the result of the increased ejaculate volume and number of sperm produced. Other studies have also shown an increase in the ejaculation performance of boars in their first year of use for insemination. However, there are substantial discrepancies in findings pertaining to the age range and scale of changes in ejaculation performance in sexually developing boars [35]. Clark et al. [35] found a rapid increase in the number of sperm in the ejaculate of boars in the period from 8–10 months to 14 months, followed by the stabilisation of the number of sperm. The results of some studies indicate that variation in the ejaculate parameters of boars used for artificial insemination is already decreasing at 12–16 months [36]. Schulze et al. [16] showed that ejaculate volume and the number of sperm in the ejaculates of boars increase up to the age of 17 months, whereas Wolf and Smital [30] and Knecht et al. [37] reported that ejaculate volume in young boars increases up to the age of 24 months. They observed the highest rate of increase in ejaculate volume up to the age of 13 months, while maximum ejaculation performance, measured as the number of sperm in the ejaculate, was attained by Landrace boars at the age of 21 months and by Large White boars at the age of 19 months. Some studies have also shown that age-related changes in ejaculate volume and the total number of sperm in the ejaculate depend on the season of the year [38].

The effect of age on ejaculate quality has also been the subject of research in other animal species [39,40,41] and in humans [42,43]. Most studies have focused on the negative consequences of progressing age in older males. Increasing age has been shown to be linked to a substantial decrease in basic semen parameters, such as ejaculate volume, total number of sperm in the ejaculate, sperm concentration and motility, and the morphology and viability of sperm [44,45]. However, in terms of use for artificial insemination the course of changes in ejaculate characteristic in young breeders that are still developing is more interesting. The present study shows that in young boars in their first year of use, these changes mean an increase in the ejaculate volume of insemination doses. However, the rate of these changes depended on the breed. The increase in ejaculate volume and the number of spermatozoa in the ejaculate was very large in Landrace and Pietrain boars, while in Duroc boars, these characteristics changed very little. Similar studies have been conducted on bulls, showing that ejaculate volume increases in the period from when the bulls achieve sexual maturity to when they reach full adulthood [46,47,48]. Ejaculate volume in bulls has also been shown to increase by about 0.5 mL each year during this period [49]. The increase in ejaculate volume in bulls during this period has been attributed to the development of the accessory sex glands, increased scrotal circumference, and the bull’s increasing body weight [50].

The results of the present study indicate that there are differences in the sexual development of boars of different breeds. Differences were demonstrated in the rate of changes in ejaculate volume and the number of sperm in the ejaculate during the first year of use for artificial insemination. The breed that stood out the most in this regard was Duroc. Ejaculate volume in boars of this breed was much lower than in the other breeds and did not increase with age (Figure 1). The number of sperm in Duroc boar ejaculates also did not increase with age, as is the case with the other boar breeds (Figure 2). Some reports based on the evaluation of very young boars have suggested that lower ejaculate volume at the start of use for artificial insemination may be due to later sexual maturation in Duroc boars. However, this conclusion does not seem justified, especially as other studies have shown low levels of basic physical ejaculate characteristic in older Duroc boars as well [20].

The present study shows no clear tendencies for the changes in sperm concentration in the ejaculates collected in the first year of the boars’ use (Table 3). Sperm concentration did not increase in a similar manner to ejaculate volume or the number of spermatozoa in the ejaculate. In the literature, reports on the effect of boar age on sperm concentration in the ejaculate do not lead to definitive conclusions. Some data indicate that sperm concentration in the ejaculate increases with the age of boars. A study using Large White boars showed that the sperm concentration in ejaculates collected from 14 to 17 months of age was significantly lower than in ejaculates collected from 18 to 29 months of age [51]. Tvrdá et al. [52] also showed a much lower sperm concentration in young boars below the age of 12 months than in older boars at the age of 24–36 months. Studies have also shown a decrease in sperm concentration as boars grow older [53,54], while Schulze et al. [16] did not confirm significant changes in sperm concentration in the ejaculates of boars collected at the age of 8–17 months.

The fertilising ability of semen depends on sperm motility [55]. The present study did not show significant changes in sperm motility in the boars’ first year of use (Table 3), confirmed in all of the breeds analysed (Figure 6). This is supported by findings presented by Banaszewska and Kondracki [13]. Hensel et al. [56], in a study conducted on Duroc and Pietrain boars, showed that sperm motility in fresh semen is higher in Pietrain boars than in Duroc boars, but they also did not confirm that sperm motility was dependent on the age of the boars. The effect of boar age on sperm motility was also not confirmed in a study by Tsakmakidis et al. [57]. Sevilla et al. [58], conversely, showed that the percentage of motile sperm was about 6–7% higher in young boars below the age of 12 months than in boars at the age 12–24 months and above 24 months. Tvrdá et al. [52] reported much lower sperm motility in young boars below the age of 12 months than in older boars aged 24–36 months. Data on the effect of boar age on sperm motility are therefore not unequivocal.

The effect of seasonal factors on semen quality can be analysed according to the criterion of the season of the year [38] or according to individual months [59]. Schulze et al. [16] reported that the season has a minor effect on boar ejaculate characteristics. The present study shows no differences in the sperm concentration, sperm motility, or volume of ejaculates collected in the spring, summer, autumn, and winter; the observations of Schulze et al. [16] are largely in agreement with these findings. Frydrychova et al. [60] also showed no significant effect of the season of the year on sperm concentration or the total number of sperm in the ejaculate. However, studies have indicated that the ejaculate characteristics of boars are affected by season [25,61,62]. Ejaculates collected in spring and summer (from April to September) have been shown to have a lower total sperm count than those collected in the autumn and winter [30,61]. The negative effect of the summer on semen quality may also depend on the environmental conditions in the piggery. The main cause is the ambient temperature. An ambient temperature above 29 °C significantly reduces the reproductive performance of boars and adversely affects semen quality [18]. Seasonal variation in the ejaculate characteristics of boars is believed to be due to the effects of the photoperiod and air temperature, as these are the main seasonal factors that can influence the course of spermatogenesis in boars [63,64]. Highest-quality ejaculates are usually obtained from boars during the period of decreasing daylight, meaning the autumn and winter, while ejaculation performance drops significantly in the spring and summer, as low blood androgen levels are observed at this time [65]. Fewer insemination doses are also obtained from ejaculates collected during the spring and summer [25].

## 5. Conclusions

In summary, variations in ejaculate characteristics mainly depend on factors associated with boar breed and age. The ejaculates of Landrace boars were the most favourable for artificial insemination. Their ejaculates have a high volume with a relatively high sperm concentration and the highest number of sperm. The highest number of insemination doses can be obtained from the ejaculates of Landrace boars—on average, 2.7–6.7 more doses than from other breeds. Duroc boars had the most distinctive ejaculate characteristics, with a very low ejaculate volume but a high sperm concentration and the highest sperm motility at the same time. The ejaculate characteristics of Pietrain boars showed the opposite pattern, with the highest ejaculate volume but the lowest sperm concentration. The rate of changes in ejaculate characteristics in the first year of use is a breed trait. Ejaculate volume and the number of spermatozoa in the ejaculates increase very rapidly in Pietrain and Landrace boars. The rate of these changes in Large White boars was much lower, and they were very slight in Duroc boars. The effect of the season of the year on ejaculate characteristics is relatively small, but autumn appears to be the most favourable season for the use of boars for artificial insemination. Ejaculates collected in autumn have the highest volume—significantly higher than ejaculates collected in spring and summer.

## 6. Practical Implications

In planning the coverage of the demand for boar semen, insemination stations should take into account the differences in the sexual development of boars of different breeds. The Duroc breed requires special attention. From the ejaculates of boars of this breed, few insemination doses are obtained not only at the beginning of their use but also later on, at ages over 17 months. In boars of other breeds, particularly in Landrace during the first year of use, there is intense sexual development, resulting in an increase in the volume of ejaculates and the number of sperm, and consequently also in the number of insemination doses obtained. This should be taken into account when planning the number of boars used at insemination stations.

## Figures and Tables

**Table 1:** The share of individual components of variability in the total variability of the physical characteristics of the ejaculate (percentage share of mean squared deviations).

Specification		Sum of Mean Square Deviations	Mean Square Deviations for Individual Sources of Variation							
			Breed	Age	Season	Breed × Season	Breed × Age	Season × Age	Breed × Season × Age	Error
Ejaculate volume	MS	148,082.84	36,244.06	52,909.97	18,000.71	7068.70	10,178.45	7130.26	9178.05	7372.65
	%	100.00	24.48	35.73	12.16	4.77	6.87	4.82	6.20	4.98
Sperm concentration	MS	150,132.58	61,289.47	5996.45	7459.13	6607.64	22,044.52	15,366.32	21,134.37	10,234.69
	%	100.00	40.82	3.99	4.97	4.40	14.68	10.24	14.08	6.82
Sperm motility	MS	143.43	18.84	12.94	13.08	18.42	18.95	24.01	19.18	18.01
	%	100.00	13.13	9.02	9.12	12.84	13.21	16.74	13.37	12.56
Total number of spermatozoa	MS	19,727.73	6425.21	5699.87	1174.08	778.17	3400.06	902.39	516.87	831.08
	%	100.00	32.57	28.89	5.95	3.94	17.23	4.57	2.62	4.21
Number of insemination doses per ejaculate	MS	2346.66	842.89	633.32	146.79	109.75	377.13	87.56	59.79	89.42
	%	100.00	35.92	26.99	6.26	4.68	16.07	3.73	2.55	3.81

**Table 2:** Effect of breed on semen traits.

Specification		Breed			
		Landrace	Large White	Pietrain	Duroc
Number of ejaculates (n)		381	354	118	90
Ejaculate volume (mL)	mean	265.33 ^a^	249.04 ^a^	277.11 ^a^	191.08 ^b^
	SD	91.07	96.92	93.02	51.68
Sperm concentration (×10^6^/mL)	mean	456.54 ^a^	435.37 ^b^	411.10 ^b^	529.23 ^c^
	SD	99.65	108.21	87.03	117.53
Sperm motility (%)	mean	77.58 ^ab^	77.59 ^ab^	76.77 ^a^	78.77 ^b^
	SD	4.28	4.34	4.69	3.29
Total number of spermatozoa (×10^9^)	mean	92.22 ^a^	81.55 ^b^	86.85 ^ab^	78.57 ^b^
	SD	34.24	29.81	25.89	20.62
Number of insemination doses per ejaculate (n)	mean	31.22 ^a^	27.51 ^b^	28.53 ^ab^	24.54 ^c^
	SD	11.26	9.47	8.84	6.68

Different superscript letters ^a, b, c^ depict significant differences between values within rows at *p* < 0.05.

**Table 3:** Effect of age on semen traits.

Specification		Age			
		≤10 months	11–13 months	14–17 months	>17 months
Number of ejaculates (n)		175	225	284	259
Ejaculate volume (mL)	mean	213.92 ^a^	241.41 ^b^	263.29 ^c^	280.38 ^c^
	SD	73.67	80.07	92.69	105.30
Sperm concentration (×10^6^/mL)	mean	459.00 ^a^	448.25 ^a^	445.79 ^a^	449.48 ^a^
	SD	98.89	107.68	106.67	113.42
Sperm motility (%)	mean	77.82 ^a^	77.64 ^a^	77.57 ^a^	77.45 ^a^
	SD	4.27	4.25	4.29	4.36
Total number of spermatozoa (×10^9^)	mean	74.04 ^a^	80.94 ^a^	88.52 ^b^	96.57 ^c^
	SD	20.99	26.18	29.94	37.19
Number of insemination doses per ejaculate (n)	mean	25.01 ^a^	27.43 ^ab^	29.63 ^b^	31.84 ^c^
	SD	7.40	8.33	9.98	12.24

Different superscript letters ^a, b, c^ depict significant differences between values within rows at *p* < 0.05.

**Table 4:** Effect of season on selected semen traits analysed in the experiment.

Specification		Season			
		Spring	Summer	Autumn	Winter
Number of ejaculates (n)		224	257	238	224
Ejaculate volume (mL)	mean	244.64 ^a^	244.84 ^a^	270.35 ^b^	254.83 ^ab^
	SD	83.08	89.22	100.62	97.34
Sperm concentration (×10^6^/mL)	mean	462.41 ^a^	448.61 ^a^	439.63 ^a^	449.54 ^a^
	SD	109.97	103.58	101.88	113.91
Sperm motility (%)	mean	77.41 ^a^	78.09 ^a^	77.56 ^a^	77.27 ^a^
	SD	4.39	3.93	4.39	4.46
Total number of spermatozoa (×10^9^)	mean	84.96 ^a^	84.35 ^a^	90.25 ^a^	85.42 ^a^
	SD	30.92	32.30	31.73	28.26
Number of insemination doses per ejaculate (n)	mean	29.12 ^a^	27.97 ^a^	29.97 ^a^	28.41 ^a^
	SD	10.19	10.61	10.55	9.11

Different superscript letters ^a, b^ depict significant differences between values within rows at *p* < 0.05.

## Data Availability

The data are available on request from the corresponding author.
